# A colorimetric strategy based on dynamic chemistry for direct detection of Trypanosomatid species

**DOI:** 10.1038/s41598-019-39946-0

**Published:** 2019-03-06

**Authors:** Mavys Tabraue-Chávez, María Angélica Luque-González, Antonio Marín-Romero, Rosario María Sánchez-Martín, Pablo Escobedo-Araque, Salvatore Pernagallo, Juan José Díaz-Mochón

**Affiliations:** 1 DestiNA Genomica S.L. Parque Tecnológico Ciencias de la Salud (PTS), Avenida de la Innovación 1, Edificio BIC, 18016 Armilla, Granada Spain; 2 GENYO Centre for Genomics and Oncological Research, Pfizer/University of Granada/Andalusian Regional Government. PTS Granada - Avenida de la Ilustración, 114– 18016 Granada, Spain; 3 Department Medicinal and Organic Chemistry, School of Pharmacy, University of Granada, Campus Cartuja s/n, 18071 Granada, Spain; 4 ECsens, CITIC-UGR, Department of Electronics and Computer Technology, University of Granada, Campus Aynadamar, 18071 Granada, Spain; 5 DestiNA Genomics Ltd., 7-11 Melville St, Edinburgh, EH3 7PE United Kingdom

## Abstract

*Leishmaniasis* and *Chagas* disease are endemic in many countries, and re-emerging in the developed countries. A rapid and accurate diagnosis is important for early treatment for reducing the duration of infection as well as for preventing further potential health complications. In this work, we have developed a novel colorimetric molecular assay that integrates nucleic acid analysis by dynamic chemistry (ChemNAT) with reverse dot-blot hybridization in an array format for a rapid and easy discrimination of *Leishmania major* and *Trypanosoma cruzi*. The assay consists of a singleplex PCR step that amplifies a highly homologous DNA sequence which encodes for the RNA component of the large ribosome subunit. The amplicons of the two different parasites differ between them by single nucleotide variations, known as “Single Nucleotide Fingerprint” (SNF) markers. The SNF markers can be easily identified by naked eye using a novel micro Spin-Tube device "Spin-Tube", as each of them creates a specific spot pattern. Moreover, the direct use of ribosomal RNA without requiring the PCR pre-amplification step is also feasible, further increasing the simplicity of the assay. The molecular assay delivers sensitivity capable of identifying up to 8.7 copies per µL with single mismatch specificity. The Spin-Tube thus represents an innovative solution providing benefits in terms of time, cost, and simplicity, all of which are crucial for the diagnosis of infectious disease in developing countries.

## Introduction

Parasitic diseases not only cause millions of deaths per year, but also have serious health and economic consequences^1^. Among all the parasitic diseases, protozoan parasites of the family *Trypanosomatidae* are responsible for devastating diseases in humans, dogs as well as livestock, resulting in severe illness or even death if left untreated^1,2^. The protozoa *Trypanosoma cruzi* (*T*. *cruzi*) and *Leishmania spp*. are the causative agents of *Chagas* disease and *Leishmaniasis* respectively^3^. Even though these diseases are endemic in tropical and sub-tropical regions of the world, in the last decades there has been a re-emergence also in developed countries, with international travel being responsible for increases in reported cases of *Trypanosomatidae* infections in non-endemic countries^1,4^. Consequently, these parasitic infections have become a more important public health issue of global relevance. Even though there are several methods available for the screening of parasitic infections, however, they have not yet changed dramatically the diagnosis in developing countries. Microscopic identification and parasite cultivation are still the primary diagnostic tools employed in many regions where *Leishmaniasis* is endemic^5^. These methods, unfortunately, require extended incubation times and are expensive, requiring specialized equipment and highly trained personnel^6^. On the other hand, standard serological approaches that could potentially prove to be timely and cost effective for diagnosing parasitic infections are of limited value, as most patients do not develop a significant antibody response^7^. In addition, and problematically for clinicians attempting to make an accurate diagnosis, *T*. *cruzi* and *Leishmania spp*. share various antigens that cause cross-reactivity in serological diagnosis when complex antigenic mixtures are used^8^. As a result, Nucleic Acid Testing (NAT) methodologies have become valuable methods for the routine and accurate assessment of parasitic infectious diseases. NAT benefits from the presence of specific nucleic acid fragments for determining the presence/absence of pathogens^3,9–13^. There are different methods ranging from probe hybridization, amplification of genomic targets and sequencing of nucleic acid fragments. The use of Nucleic Acid Amplification Techniques (NAATs) such as Polymerase Chain Reaction (PCR), real-time PCR or Nucleic Acid Sequence Based Amplification (NASBA) can be very useful in detecting infections and post-treatment monitoring^9,13,14^, including detection and distinguishing of parasites using single base resolution methods such as Single Base Extension (SBE). Primers are designed to hybridize to a complementary nucleic acid region such that the 3′ end of the primer finishes immediately before the nucleotide under interrogation^15^. However, while NAT has the advantage of being sensitive, there are some limitations, as the NAT methodologies have complex protocols, require technical expertise to run and interpret data (not cost-effective), and utilize equipment that is incompatible with use in remote and low-resource locations, such as developing countries where diagnostic laboratories are often poorly resourced and sparsely distributed^16^. Thus, there is an urgent need for early detection of infectious disease with improved, simple and low-cost alternative tests not requiring expensive laboratory equipment to be performed. To sum up, while laboratory-based models require benchtop laboratory facilities and complex procedures, Point-of-Care testing allows a more decentralized diagnostic analysis with additional advantages such as portability, automation, shorter time-to-results and lower cost^17–19^. The World Health Organization (WHO) is consistently encouraging developers to implement these new diagnostic technologies for improved effective clinical management and treatment of the major infections suitable in developing countries context^20,21^. These diagnostic devices should follow WHO criteria, the ASSURED acronym (affordable, sensitive, specific, user-friendly, rapid and robust, equipment-free and deliverable)^22,23^. Following this encouragement, Zhang *et al*. recently developed a low-cost CRISPR-based diagnostic (CRISPR-Dx) for detecting DNA or RNA molecules of pathogenic species with single mismatch specificity^24,25^.

Besides the tests available today^19,26^, our group has recently reported the successful detection and differentiation of three *Trypanosomatids* species, by detecting Single Nucleotide Fingerprint (SNF) markers using a dynamic chemical approach for nucleic acid reading (ChemNAT technology)^27,28^ in combination with MALDI-ToF^29^. SNF markers are single nucleotide variations that occur at specific positions in conserved target nucleic acid sequences, allowing the differentiation of pathogenic species. As a result, these SNF represents the perfect target to be interrogated by the ChemNAT technology through the specific dynamic incorporation of aldehyde-modified SMART-Nucleobases into the abasic position of abasic PNA probes. Despite its high specificity, the use of this method has limitations, with higher cost and complex instrumentation (MALDI-ToF) required, making this approach unsuitable for diagnosis of parasitic infections in developing countries.

**Figure 1:**
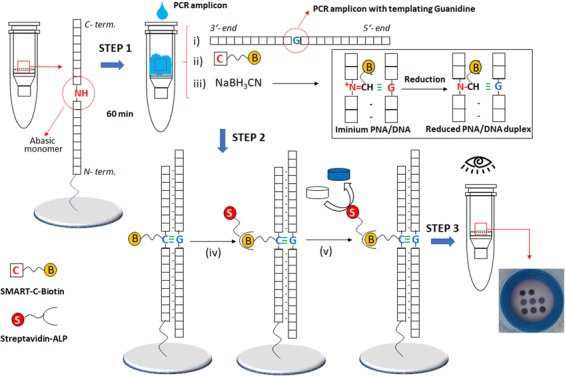
Spin-Tube assay: Merging of dynamic chemistry with a simple colorimetric end-point assay in a novel micro Spin-Tube device for analysing “Single Nucleotide Fingerprint” (SNF) markers. Step 1: modification of abasic PNAs, which are immobilized onto nylon membranes following specific spot patterns, with the biotinylated aldehyde-modified cytosine (SMART-C-Biotin). This process requires three steps: (i) perfect hybridization between abasic PNAs and PCR amplicons; (ii) generation of a reversible iminium specie between the secondary amine of the “abasic” unit and the aldehyde group of the SMART-C-Biotin nucleobase driven by the templating nucleobase. In this case, SMART-C-Biotin incorporation can be just templated by a guanidine as otherwise the iminium specie is not stable enough to be reduced; and (iii) reduction of the iminium specie by sodium cyanoborohydride to yield a non-reversible tertiary amine within the PNA printed onto the nylon membrane. Step 2: (iv) Biotin labelling with streptavidin alkaline phosphatase (Streptavidin-ALP); (v) Incubation with the chromogenic substrate (NBT/BCIP, nitro blue tetrazolium chloride/ 5-Bromo-4-chloro-3-indolyl phosphate) which generates a blue precipitate. Step 3: Data analyses by naked-eye reading. Time for the assay: 90 min PCR amplification; 60 min dynamic chemistry reaction; 30 min color development procedure, ±3 h total).

Here, we report the adaptation of our previous method onto a colorimetric reverse dot-blot^30–35^ assay that has the potential to fulfil the WHO ASSURED criteria, for the diagnosis of *Leishmaniasis* and *Chagas disease*. This novel method combines ChemNAT technology with a simple colorimetric end-point assay on a porous nylon membrane contained within a micro Spin-Tube device (Spin-Tube). The assay uses a singleplex PCR to amplify a highly conserved sequence of DNA, which encodes the RNA component of the large ribosome subunit with SNF markers for the two different parasite species under interrogation. This amplicon is later interrogated by the ChemNAT technology that relies on a dynamic chemical approach for nucleic acid testing. As shown in Fig. 1, the SNF sequence analysis is based on combining biotinylated aldehyde-modified cytosine (SMART-C-Biotin) with unique abasic PNA probes to target PCR amplicon strands, such that a nucleobase-free position on the PNA strand (known as ‘abasic’ position) lies opposite to two different nucleotides under interrogation on the DNA amplicons. A reversible reaction between the SMART-C-Biotin and a free secondary amine on the abasic PNA probes generates an iminium intermediate, which can be chemically reduced to a stable tertiary amine. Within the novel Spin-Tube, the dynamic chemistry reaction mixture is added to the nylon membranes on which the abasic PNA probes have been immobilized following a specific spot pattern (Fig. 1, Step 1). The PCR amplicons act as template molecules and drive the specific incorporation of SMART-C-Biotin molecules into a specific abasic PNA according to Watson-Crick DNA base pairing model in which the SMART-Cytosine, in this case carrying a biotin tag, is ONLY recognized by a guanidine nucleotide (Fig. 1, Step 2). The labelling is then achieved by streptavidin alkaline phosphatase (Streptavidin-ALP) to produce colorimetric signal (blue precipitate) patterns when the chromogenic substrate (NBT/BCIP, nitro blue tetrazolium chloride/5-Bromo-4-chloro-3-indolyl phosphate) is added. Each parasite gives a unique colored spot pattern that can be read by naked-eye (Fig. 1, step 3).

## Results and Discussion

### Conserved region amplification and abasic probes design

**Figure 2:**
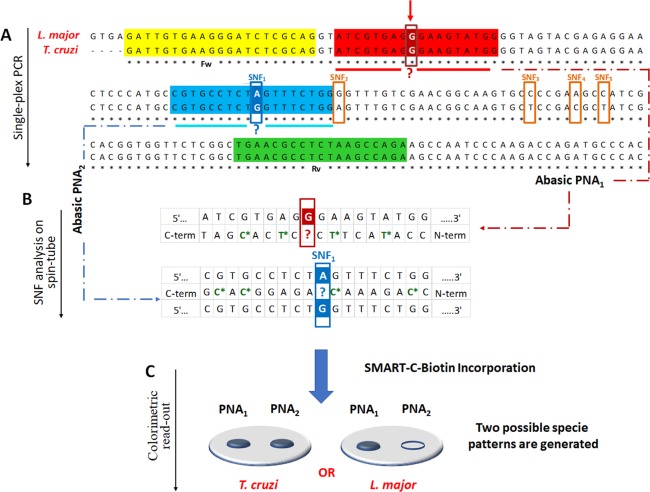
(**A**) Multiple sequences alignment of *L*. *major*vs *T*. *cruzi*. Forward (highlighted in yellow) and reverse (highlighted in green) primers. Respectively, highlighted in red and blue, the abasic PNA_1_ and PNA_2_ complementary region in the PCR amplicon. Sequence alignment shows 5 SNFs (emphasized with orange rectangles). A red arrow indicates the guanidine “G” nucleotide at +32 from the 5′-terminus under interrogation *via* abasic PNA_1_ probe (emphasized also with a red rectangle), while the abasic PNA_2_ probe interrogates the first SNF (SNF_1_ emphasized with the blue rectangle). (**B**) Schemes of region of the PCR amplicons interrogated using the two abasic PNA probes. The chiral PNA monomers are identified with a star and highlighted in green. Incorporation of SMART-C-Biotin provides proof-reading, indicating the presence of the parasitic genome, as well as the discrimination between *L*. *major* vs *T*. *cruzi*. The white letters within the red and blue squares indicate the templating nucleotides that lie opposite the abasic position of PNA_1_ and PNA_2_, represented with the red or blue question mark respectively. (**C**) Each parasite gives rise to a specific SMART-C-Biotin incorporation pattern. *T*. *cruzi* gives a positive result for both abasic probes, whereas *L*. *major* only shows a positive result in one, abasic PNA_1_.

PCR amplification was carried out to amplify the conserved region encoding for the 28S ribosomal RNA delta genes. As shown from the multiple sequence alignment in Fig. 2A, the PCR amplification of the target sequence was performed using a single pair of primers (singleplex PCR) which amplifies any of the parasite species present in the sample. The target region is highly conserved among the *Trypanosomatids* *T*. *cruzi* and *L*. *major*, except for five SNFs. Amplified fragment analysis was performed using the previously described Spin-Tube. Briefly, abasic PNA probes, PNA_1_ and PNA_2_ were designed in order to hybridize efficiently with the amplified single strand sense DNA, with the important particularity that the abasic positions in the PNA probes are opposing the nucleobases under interrogation. Once the abasic PNA probes hybridize with target sequences, the SMART-C-Biotin dynamic incorporation takes place, enabling the unequivocal identification of the parasite present in the amplicon sample, because of the unique colorimetric pattern that each *Trypanosomatid* amplicon generates. As shown in Fig. 2B, the abasic PNA_1_ probe was designed so that its abasic site lies opposite a guanidine nucleotide (at position + 32 of the amplicon), irrespective of the species, so as to give a positive result for both parasites, thus confirming the presence of one or both *Trypanosomatids* in the sample (see red arrow in Fig. 2A). At the same time, the abasic PNA_2_ is the probe that enables discrimination of the *Trypanosomatid* species. As the target parasite *T*. *cruzi* has a guanidine at the position that lines up with the “abasic” position in the abasic PNA_2_ probe (SNF_1_ in Fig. 2B), the SMART-C-Biotin incorporation is templated, thereby covalently incorporating the biotin tag into the probe attached to the membrane, resulting in a blue spot after applying the labelling and color development protocol. DNA coming from *L*. *major* has the same sequence but with an adenosine at the SNF_1_ position (instead of a guanine, see Fig. 2B). This DNA hybridizes with PNA_2_ but SMART-C-Biotin is not incorporated and so no signal is detected on abasic PNA_2_. In summary, this approach requires two specific molecular events to create a signal: (i) perfect hybridization between nucleic acid strands and complementary abasic PNA probes; and, (ii) specific molecular recognition, through guanidine-cytosine base-pairing to allow SMART-C-Biotin incorporation onto the abasic site of the PNA probes (Fig. 2C).

### Spin-Tube fabrication

**Figure 3:**
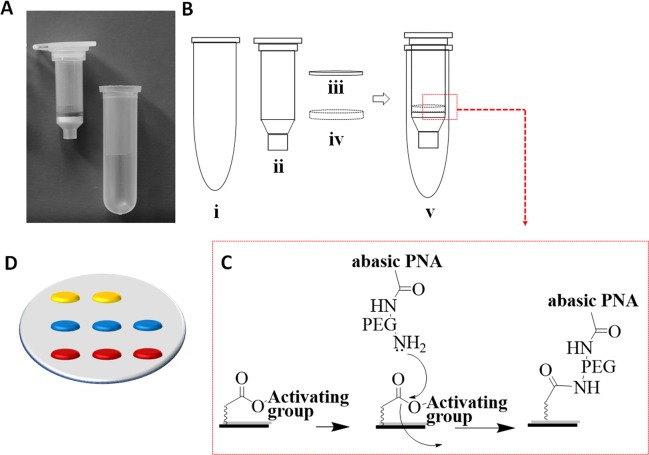
(**A**) Spin-Tube prototype; (**B**) Plastic components for the Spin-Tube fabrication (Fig. S1 in SI); (**C**) Amide formation between pre-activated carboxylic acid groups of nylon membranes and primary amine groups of abasic PNAs; (**D**) Graphic layout of the array: in yellow, 2 spots of biotin markers; in blue, 3 spots of abasic PNA_2_; in red, 3 spots of abasic PNA_1_.

The ChemNAT technology with its colorimetric reverse dot blot assay (colorimetric ChemNAT assay) was integrated into a novel micro device, known as  “Spin-Tube” (Fig. 3A). As shown in Fig. 3B, the Spin-Tube consists of: (i) a centrifuge collection tube; (ii) an internal column for the assay; (iii) a nylon membrane (pre-spotted with abasic PNA probes) immobilized onto the bottom of the column *via* a plastic pressure ring (iv). The abasic probes were amino-PEGylated at their N-terminal end and printed onto nylon membranes containing pre-activated carboxyl groups (Fig. 3C). An important stage in the construction of the Spin-Tube, apart from the immobilization of abasic PNAs, was the definition of the array probe layout (Fig. 3D). Probes with fixed concentrations were printed with an automatic nano-plotter onto the nylon membrane, and optimization of signal strength and best signal to background ratios was undertaken. Taking into account the 8 mm membrane diameter, it was decided to create a 2 × 3 array. Abasic PNA_1_ and PNA_2_ were printed onto two parallel rows of three spots each (6 features in total, in red and blue in Fig. 3D respectively). Two control biotin-labelled DNA oligomers were printed on the top row of the array, to identify array orientation and provide a labelling internal control (in yellow in Fig. 3D). Following printing, performance of probes was initially checked by using synthetic mimic DNA oligomers and SMART-C-Biotin incorporation (data not shown).

### Abasic PNA probes design and synthesis

Abasic PNA_1_ and PNA_2_ were synthesized with amino-PEGylated groups in order to be covalently bonded and immobilized onto the solid substrate (nylon membranes). As previously described by our group, abasic neutral PNA probes immobilized onto solid surfaces have been found to lack stability and can exhibit a degree of undesirable deformation, flexing and/or bending^36^. This affects the performance (e.g., specificity and/or sensitivity) of the abasic PNA probes in this assay – preventing maximum target binding and assay performance. Abasic PNA_1_ and PNA_2_ were designed and synthesized with these solid surface challenges in mind, adding PNA monomers containing propanoic acid residues at their gamma positions across the probe backbone, improving the probe performance and reducing self-aggregation (Fig. 2B and Fig. S2 in SI). Moreover, these gamma modifications give rise to a sterocenter, hence creating chiral PNAs molecules, being just the L-PNA monomers the ones producing PNA oligomers capable of hybridizing complementary natural nucleic acids^37–40^. The result was that the L-propanoic abasic PNA probes bound to nylon membrane were more readily available to hybridize to complementary nucleic acid strands. Still unknown was if such abasic PNA probes would prove to be less prone to flexing, bending or otherwise deforming from their “normal” linear configuration^36^. The modifications introduced led to the two probes demonstrating improved specificity and improved sensitivity towards a base complementary to the nucleobase of the DNA amplicon to be characterized. The abasic position monomer was also modified with this propanoic acid chain at gamma position and with the same stereochemistry allowing a better dynamic incorporation of the SMART-C-Biotin because of the spatial orientation of the free secondary amine of the “blank” position (Fig. S2 in SI).

**Figure 4:**
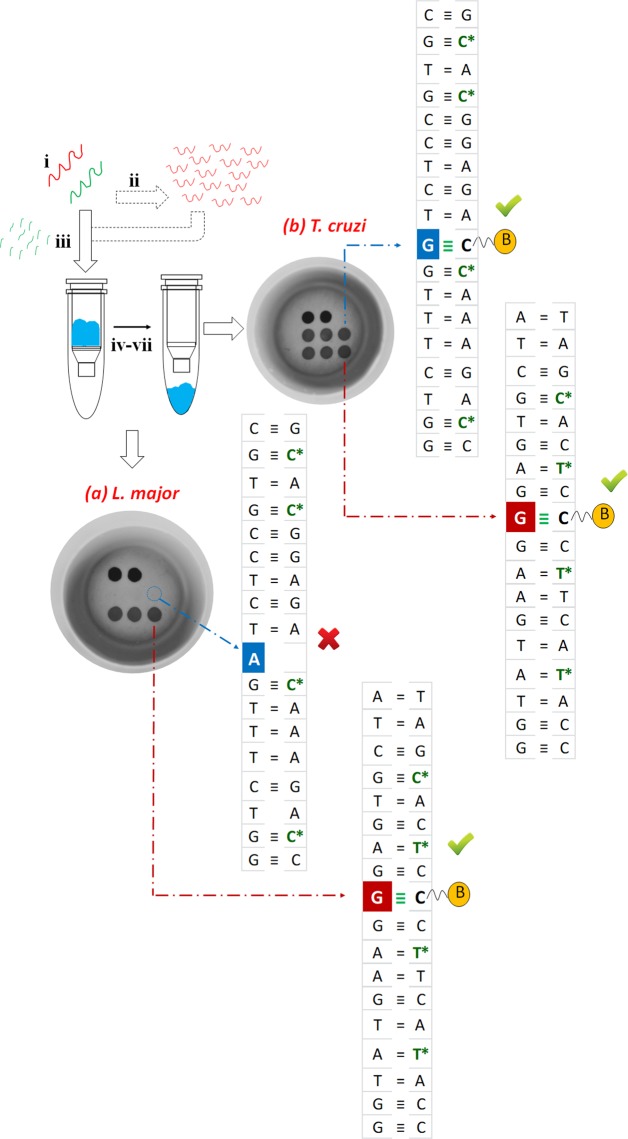
Steps of the Spin-Tube assay: (i) Samples collection (minutes) and nucleic acids extraction, either DNA (red) or RNA (green) (5 minutes); (ii) Singleplex PCR amplification of gDNA (90 minutes); (iii) Total RNA fragmentation (15 minutes); (iv) Biotin labelling by dynamic chemistry reaction. The reaction is carried out at 45 °C inside the internal column for the assay of Spin-Tube (60 minutes); (v) Washing with centrifuge (6000 rpm) and SMART-C-Biotin labelling with streptavidin-ALP (5 minutes); (v) Washing with centrifuge (6000 rpm) and addition of colorless chromogenic reagent to form a blue precipitate (8 minutes); (vi–vii) Washing and drying with centrifuge enable an unequivocal identification of the parasite present in the sample. Each parasite gives a unique colorimetric pattern: respectively (a) and (b) spot patterns for *L*. *major* and *T*. *cruzi*.

### Labelled SMART Nucleobase for the colorimetric detection

While our previous mass spectrometry method^29^ allowed parasite species detection and characterization by measurement of molecular weight differences between abasic PNA probes and aldehyde-modified nucleobases (SMART-Bases), in this Spin-Tube platform it was required that the SMART-Base were biotin-labelled (Figs S3–S5 in SI)^41,42^. Here the color development reaction depends on the biotin recognition by a streptavidin-alkaline phosphatase complex, that transforms a colorless chromogenic substrate (NBT/BCIP, nitro blue tetrazolium chloride/5-Bromo-4-chloro-3-indolyl phosphate) into a blue precipitate each time there is a biotin tag attached to the membrane. The blue spots emerging correspond to those abasic PNA probes which have successfully hybridized with the complementary PCR products and in whose abasic positions SMART-C-Biotin has been incorporated through a guanosine template (Fig. 2). The SMART-C-Biotin bears a PEG spacer to increase water solubility while at the same time distancing the nitrogenous base involved in the hydrogen bonding recognition from the biotin tag responsible for the color development^43^.

### Sequence analysis of genomic DNA

The Spin-Tube platform was validated using PCR products from genomic DNA (gDNA) of both parasites. Singleplex PCR was carried out to amplify the highly conserved segment of DNA containing the two single nucleotides under interrogation (Fig. 2). DNA amplicon products were denatured and then together with the dynamic chemistry reaction reagents added directly into the internal column of the Spin-Tube that supports the nylon membrane for the color-development assay (Fig. 4). Subsequently, the reaction was carried out at a constant temperature of 45 °C avoiding the need to make use of sophisticated apparatus. Single stranded DNA hybridizes with the abasic PNAs acting as template molecules, driving the error free incorporation of SMART-C-Biotin into the specific chemical pocket. Finally, the incorporated SMART-C-Biotin is labelled with a streptavidin-ALP, so that a colorimetric signal pattern was generated when the chromogenic substrate is added. The signal pattern generated by the protocol allows the visual or photographic imaging of which species cause the patient infection. *L*. *major* gDNA generated a signal ONLY at the abasic PNA_1_ probe, as its abasic site lies opposite a guanidine at +32 position from the 5′-terminus of amplicon (see red arrow in Fig. 2). While, *T*. *cruzi* gDNA generated signals for both abasic PNA probes. The abasic sites of both PNA probes lie opposite a guanidine, allowing the incorporation of SMART-C-Biotin with its biotin tag, thus resulting in a blue spot after its incorporation. Results in Fig. 4 show a successful identification of parasites present in samples, and in line with the expected results, showing (a) *L*. *major* positive for abasic PNA_1_ ONLY and (b) *T*. *cruzi* positive for both abasic PNA probes.

### Assay specificity

**Figure 5:**
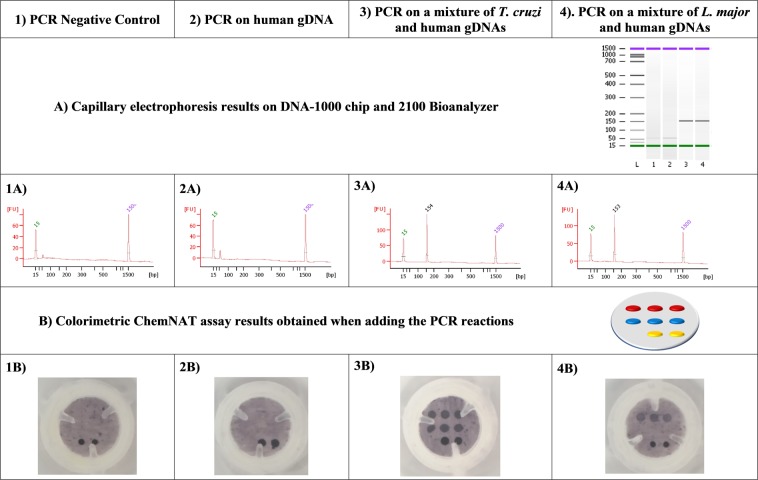
(**A**) Capillary electrophoresis and (**B**) colorimetric results. First row shows the template used for the PCR reaction (1 to 4). Second and third rows show the capillary electrophoresis results (1A to 4A). Fourth and fifth rows show the colorimetric results (1B to 4B). Regarding columns: Column 1 corresponds to the PCR negative control (water) showing the absence of PCR contamination (1A) and the look of the membrane after all the procedure with just the biotin positive control of the colorimetric assay (1B). Column 2 shows the PCR performed on 20 ng of human gDNA highlighting the specificity of the previously designed primers (2A and 2B). Column 3 matches with the PCR performed on 20 ng of *T*. *cruzi* gDNA sample that has been mixed with an equal amount of human gDNA (3A and 3B) and column 4 shows the experiment carried out on 20 ng of *L*. *major* gDNA samples mixed with other 20 ng of human gDNA (4A and 4B).

Bioinformatic along with experimental analysis were carried out to discard the possibility that the presence of human gDNA on the samples could affect the singleplex PCR. Initially, a primer BLAST study was carried out using the NCBI primer BLAST tool and no human hits came out. Primers specificity against human gDNA was experimentally checked. 20 ng of human gDNA were used as PCR template for its amplification with the set of primers described in our study and neither colorimetric signals nor bands were detected using the Spin-Tube and capillary electrophoresis analysis respectively (Fig. 5, column 2: 2A and 2B), hence probing the specificity when human gDNA is present. We also mixed 20 ng of human gDNA with 20 ng of DNA from both *Trypanosomatid* species in order to perform PCR and amplicon analysis by capillary electrophoresis and Spin-Tube approaches (Fig. 5, column 3: 3A and 3B and column 4: 4A and 4B). It was confirmed that amplicon formation of the *Trypanosomatid* species and their detection were not affected by the presence of the human gDNA.

In order to clarify the specificity of the PCR towards other microorganisms, an *in silico* study was carried out (data not shown). 28S ribosomal RNA delta gene sequences from *Leishamania spp*., *Trypanosoma brucei* and *Trypanosoma cruzi* were aligned using EMBL-EMI tool for multiple sequence alignment Clustal Omega to check that not only the primers but also the designed abasic PNA probes were suitable for the Spin-Tube and MALDI-ToF approaches. This tool was also used to study which other *Leishmania* species were suitable to be submitted to this singleplex PCR. Forward and reverse primers were blasted towards other organisms using NCBI primer BLAST tool. It was found that mainly genome from *Trypanosomatids* species matched the designed primers. Although, a few other genome sequences could also be amplified by our designed primers (*Schistosoma mansoni*, *Leptomonas pyrrhocoris*, *Aspergillus sclerotioniger* and *Aspergillus eucalypticola*), all these infectious agents cause diseases with clinical manifestations very different from those manifested in *Leishmaniasis* and *Chagas* disease and therefore other diagnostic paths would be proposed. Another key factor of the assay Spin-Tube is the signal emerges after the dynamic incorporation of SMART-C-Biotin into the abasic site of the abasic PNA probes. Even if off-target sequences are amplified, no colored spots will be observed. So, DNA strands coming from any off-target species will not create a signal, as that DNA strends will not have precise homology with both forward and reverse primers, as well as the region complementary to the abasic PNA within a narrow area of its genome.

### Assay sensitivity

**Figure 6:**
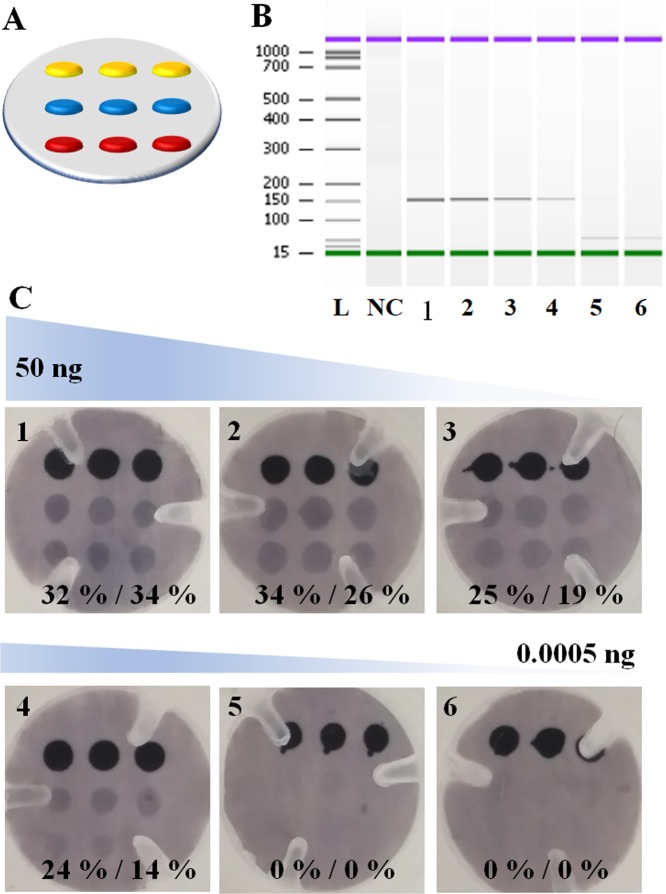
(**A**) Spotting layout with 3 Biotin marker spots (yellow) rather than the 2 of Fig. 3D. 3 spots of PNA_1_ (red) and 3 spots of PNA_2_ (blue). (**B**) Agilent Bioanalyzer 2100 gel-like images extrapolated from the capillary electrophoresis for the PCR products of *T*. *cruzi*. These gel-like images are produced from the chromatogram of the capillary electrophoresis by the bioanalyzer analysis software. NC: negative control PCR (water); 1–6 PCR reactions using decreasing amounts of gDNA of *T. cruzi*. 1: 50 ng; 2: 5 ng; 3: 0.5 ng; 4: 0.05 ng; 5: 0.005 ng; 6: 0.0005 ng. (**C**) Decreasing amounts of PCR products were used as templates for the dynamic chemistry reaction to incorporate the SMART-C-Biotin. Positive signals were obtained on both abasic PNA probes with PCR starting concentration from 50 ng up to 0.05 ng (8.7 copies/µL) of template what coincides with the last PCR product that was able to be detected by capillary electrophoresis. A percentage of the relative intensity was calculated using as 100% signal the average of the three biotin marker spots.

Clinically relevant sensitivity was achieved using the amplification and detection platform. The assay was challenged by interrogating different PCR amplification reactions, using decreasing amounts of *T*. *cruzi* gDNA as starting material. Important to note in this study, 3 biotin markers were printed on the top row of the array, rather than 2 (Fig. 6A). Instead of simple circular plastic-ring (see red arrow in Fig. S1-D in SI), here for the construction of Spin-Tube, pressure plastic-ring with three plastic teeth (see white arrow in Fig. S1-D in SI) were used. Six different amounts of gDNA (50 ng, 5 ng, 0.5 ng, 0.05 ng, 0.005 ng, 0.0005 ng) were studied (Table S1 in SI). PCR amplifications were validated and quantified by capillary electrophoresis. All but the two lowest amounts of gDNA (0.005 ng and 0.0005 ng) produced amplicons which were able to be detected and quantified by capillary electrophoresis (Fig. 6B). The amplification products were then used as template for the dynamic incorporation of SMART-C-Biotin within the Spin-Tube. Positive signals were obtained for both abasic PNAs as anticipated, confirming the presence of *T*. *cruzi* (Fig. 6C).

A relative quantification of the signal intensity was carried out using ImageJ software. Average background signal intensity was taken and subtracted from all the measurements. Biotin signal intensity (marker) was used as reference representing 100% intensity. The signal intensity for each abasic PNA probe was extracted and expressed as a percentage of the biotin signal. Almost no difference was observed by eye for the membranes in which 50 and 5 ng of *T*. *cruzi* gDNA was used as template for the PCR, whereas the relative signal intensity quantification by ImageJ software showed a slight lowering of signal as the genomic amounts decreased (Fig. 6C). The lowest point which could be detected was 0.05 ng or 8.7 copies/µL of template (see SI, Table S1 for the correlation of ng of gDNA and copy number). No results were observed for the two lowest genomic quantities, respectively 0.05 and 0.005 ng. These results confirm what had been already observed using capillary electrophoresis, further confirming that the limit of detection of the dynamic incorporation assay depends on the PCR yield to provide sufficient template. Reactions in which not enough copies of amplicons were created were mis-called as parasite free, being false negatives. Negative control (made with H_2_O as template for the PCR) was also called as parasite free, being a true negative result.

### Analysis of spiked parasite gDNA in blood

**Figure 7:**
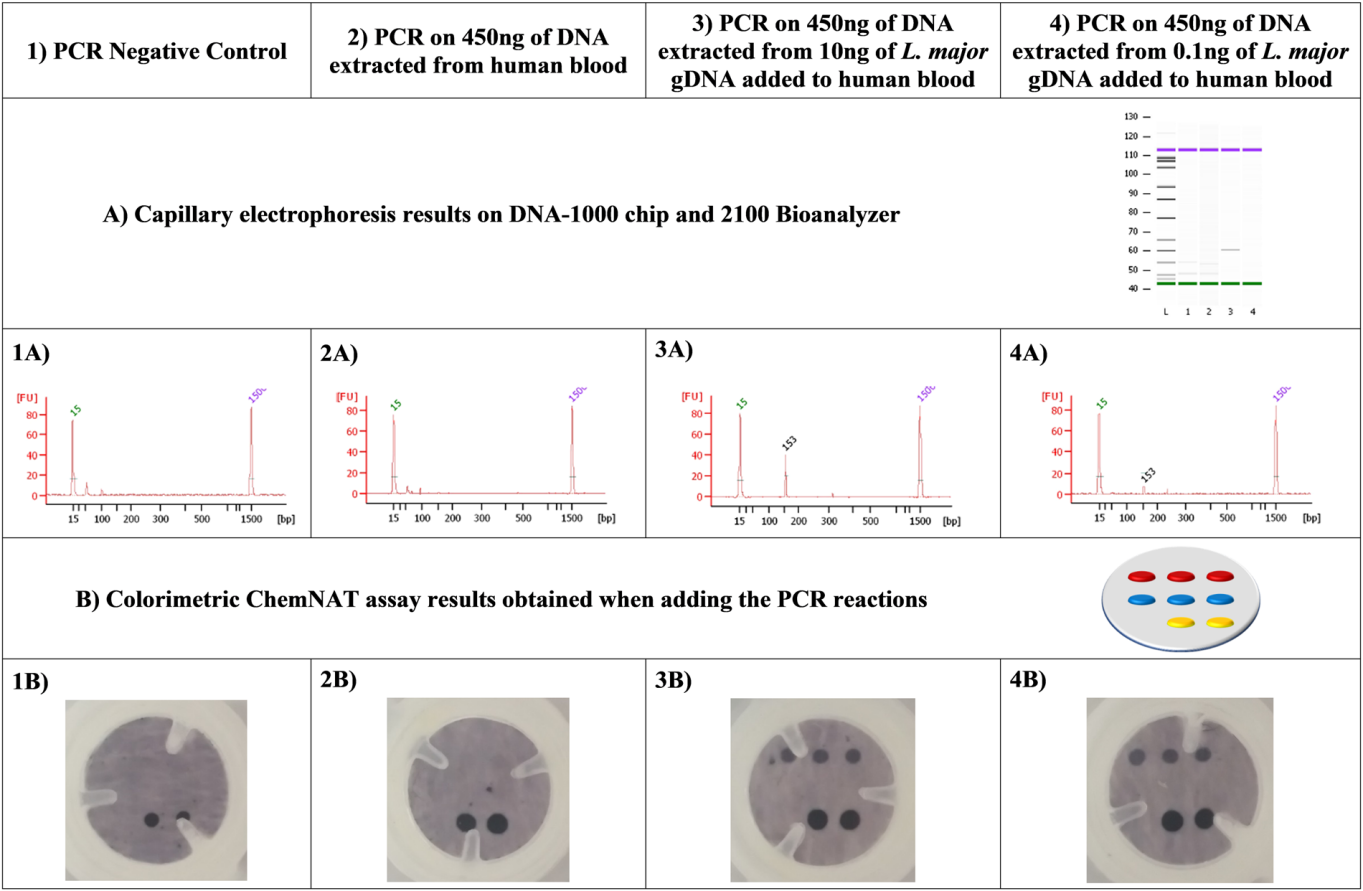
Analysis of spiked parasite gDNA in blood. (**A**) Capillary electrophoresis and (**B**) Spin-Tube results. First row indicate the template used for the PCR reaction (1 to 4). Second and third rows show the capillary electrophoresis results (1A to 4A). Fourth and fifth rows show the Spin-Tube results (1B to 4B). Regarding columns: Column 1 corresponds to the PCR negative control showing the absence of contamination (1A) and the look of the membrane after all the colorimetric procedure with just the biotin positive control of the colorimetric assay (1B). Column 2 shows the PCR performed on 450 ng of DNA extracted from 200 µL of human blood which highlights the specificity of the previously designed primers and the absence of false negatives due to the presence of human blood and DNA (2A and 2B). Column 3 contains the PCR performed on 450 ng of DNA extracted from 200 µL of human blood spiked-in with 10 ng of *L*. *major* gDNA (3A and 3B) and column 4 shows the results of the experiment carried out with a PCR performed on 450 ng of DNA extracted from 200 µL of human blood spiked-in with 0.1 ng of *L*. *major* gDNA (4A and 4B).

Verma *et al*. in their previous study^44^ reported a mean *Leishmania* parasite load of 8,372 parasites/mL in a group of 29 visceral leishmaniasis patients. In order to determine if the sensitivity of the Spin-Tube is able to detect the levels of infections reported by Verma *et al*., a spike-in experiment was performed. Two human blood samples were spiked-in with 10 and 0.1 ng of *L*. *major* gDNA and total DNA was extracted. Those extracted DNAs were used as template for PCR amplifications and the generated amplicons were analyzed by capillary electrophoresis (Fig. 7A) and by the Spin-Tube (Fig. 7B). The results confirmed that the Spin-Tube is able to detect a minimum of 0.1 ng of *L*. *major* gDNA spiked-in 200 μL of whole blood that equals to 5,000 parasite per mL of blood according the previous publication^44^.

### Direct sequence analysis of ribosomal RNA

**Figure 8:**
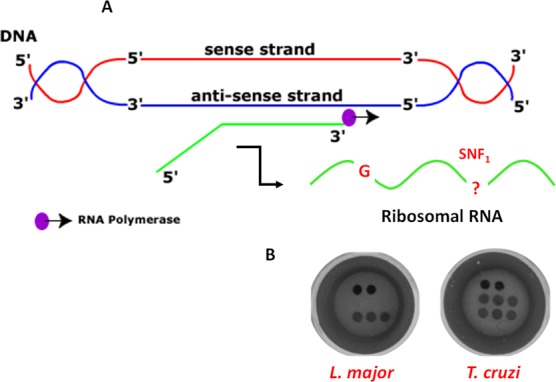
(**A**) Scheme for the synthesis of ribosomal RNA (sense RNA). The sense RNA is transcribed from the anti-sense DNA strand by RNA polymerase. The sense RNA strand templates the dynamic chemistry reaction containing both the guanidine “G” and the SNF_1_ for templating the SMART-C-Biotin incorporation respectively in the abasic PNA_1_ and PNA_2_ probes. (**B**) Unique colorimetric patterns both for *L*. *major* and *T*. *cruzi*.

Since the target nucleic acid of the assay was the sense strand DNA of the gene coding for the 28S rRNA delta unit, RNA could also be used as a target nucleic acid using the dynamic chemistry approach (Fig. 8A). Recently, the dynamic chemistry approach was used to quantify circulating miRNAs^41^, demonstrating its feasibility to detect both DNA and RNA molecules. Direct detection of ribosomal RNA would avoid performing DNA pre-amplification steps by PCR and denaturation and could significantly simplify and shorten the protocol. This would allow its implementation in developing and resources-limited countries.

A proof-of-concept study was carried out using RNA as the templating nucleic acid. Total RNA from both parasites *L. major* and *T. cruzi* was used. RNA quality and quantitation were determined using an Agilent 2100 Bioanalyzer and RNA 6000 Nano kit (Fig. S7 in SI). Total RNA was fragmented enzymatically, breaking down RNA strands into small segments to facilitate the subsequent hybridization with complementary abasic PNA probes. RNA fragmentation was checked using RNA Pico kit on an Agilent 2100 Bioanalyzer as shown in SI, Fig. S8. Dynamic incorporation of SMART-C-Biotin was performed using 10 µM of SMART-C-Biotin and 1 mM of sodium cyanoborohydride for 1 hour. The results obtained coincided with those obtained when using PCR products. *L. major* gave positive results for PNA_1_ and *T. cruzi* provided a positive signal for both PNA probes (Fig. 8B). This was a breakthrough achievement, demonstrating for the first time that direct, PCR free parasite identification from RNA samples was achieved. In addition, a much shorter protocol was developed while at the same time reducing the possibility of cross-contamination. This demostrated  that RNA can also be considered as a biomarker source for the ChemNAT approach and a future application of the Spin-Tube.

## Material and Methods

### General

All chemicals were obtained from Sigma Aldrich and used as received. SCD buffer was prepared from 2x saline sodium citrate (SSC) and 0.1% sodium dodecyl sulphate (SDS) with the pH adjusted to 6.0 using HCl. All synthetic DNA oligomers (desalted) were purchased from Microsynth AG (Balgach, Switzerland). The two abasic PNA probes (Abasic PNA_1_ and PNA_2_) were synthesized by DestiNA Genomica SL (Spain) using standard solid-phase synthesis techniques on an Intavis Bioanalytical Instruments MultiPrep CF Synthesizer (Intavis AG GmH, Germany). Aqueous solutions of abasic PNA probes were prepared and concentrations were determined using a NanoDrop ND-1000 spectrophotometer version 3.3.1. (Thermo Fisher Scientific) using as extinction coefficient values (Ɛ_260_) either 6.6, 8.8, 13.7 and 11.7 (mM^−1^ cm^−1^) for C, T, A, G, respectively. SMART-C-Biotin was prepared by DestiNA Genomica SL (Spain) as reported elsewhere^28^. Buffers and dilution reagents were provided by Master Diagnostica SL (Spain). The composition of reagents I to VI is property of Vitro SA (Spain).

### Genomic DNA samples

Parasitic protozoa genomic DNAs (gDNAs) were purchased from the American Type Culture Collection (ATCC). Respectively, gDNA from *T*. *cruzi* strain Tulahuen with ATCC ID 30266D and gDNA from *L*. *major* with ATCC ID 30012D. Human gDNA was extracted from MUM-2B cell line provided by J.C. Rodríguez-Manzaneque’s laboratory.

### Target nucleic acid selection

Multiple sequence alignment of the 28S rRNA delta genes of *L*. *major vs*. *T*. *cruzi* was carried out using the Clustal Omega, a free on-line available multiple sequence alignment tool offered by EMBL-EBI.

BLAST studies were carried out with the NCBI primer BLAST tool (https://www.ncbi.nlm.nih.gov/tools/primer-blast/primertool.cgi?ctg_time=1534265329&job_key=NT_qawTpCUEufxN6Hho3SGQBJnpJEj1nSA).

### PCR Amplification

Alignment results were used to design a single pair of primers able to amplify the target region of both parasites. Primers (5'-3') sequences were: Forward: GATTGTGAAGGGATCTCGCAG and Reverse: TCTGGCTTAGAGGCGTTCA. PCR amplification was performed on a Veriti 96-well Thermal cycler (Thermo Fisher Scientific). Cycling conditions for PCR were as follows: (1) initial denaturation at 96 °C for 3 min; (2) 40 cycles of (a) denaturation 96 °C for 30 sec, (b) annealing at 61 °C for 30 sec, and (c) extension at 72 °C for 30 sec; (3) final extension at 72 °C for 10 sec; (4) final hold at 4 °C. 5 µL of gDNA solutions containing different amounts of gDNA solution for *L*. *major* or *T*. *cruzi* were amplified using 1X PCR master mix (Thermo Fisher Scientific), 0.15 µM forward and reverse primers per reaction with a final volume of 50 µL. DNA templates were replaced with water for negative controls. PCR reactions were confirmed by capillary electrophoresis using the Agilent 2100 Bioanalyzer and DNA 1000 Kit.

### RNA fragmentation

RNA was extracted from parasites as described elsewhere^45^. RNA quality assessment and quantitation were determined using the Agilent 2100 Bioanalyzer and RNA 6000 Pico Kit (Fig. S7 in SI). NEBNext Magnesium RNA Fragmentation Module Protocol was used to fragment RNA. The following reagents were mixed in a sterile PCR tube: 1–18 µL of purified RNA containing 2–50 µg of total RNA, 2 µL of RNA fragmentation buffer and complete with nuclease-free water up to 20 µL. The mixture was incubated at 94 °C for 5 minutes (to get fragments of around 200-mer length). Then the tube was transferred to ice and 2 µL of RNA Fragmentation stop solution were added. The fragmented RNA was cleaned up using ethanol precipitation: 22 µL of fragmented RNA, 2 µL of 3 M sodium acetate pH 5.2 and 60 µL of 100% ethanol. The mixture was incubated at -80 °C for 30 minutes and then centrifuged at 14,000 r.p.m. for 25 minutes at 4 °C and ethanol was removed carefully. The pellet was washed with 300 µL of 70% ethanol, centrifuged and removed the ethanol. Finally, the pellet was air-dry for up to 10 minutes at room temperature and re-suspended in 13.5 µL of nuclease-free water. To assess the yield and size distribution of the fragmented RNA, 1 µL of a 10-fold dilution was analyzed using the Agilent 2100 Bioanalyzer and RNA 6000 Pico Kit analysis (Fig. S8 in SI).

### Abasic PNA probes spotting

An automatic immobilization of the probes on the membranes was done using Personal Arrayer 16 (CapitalBio Corporation, China). Immunodyne ABC Membrane was purchased from Pall Corporation (US). Abasic PNA probe spotting solutions were prepared to have the following final reagents concentrations 0.2025 mg/mL amaranth dye, 0.125 M sodium bicarbonate, 30% of DMSO and 15 µM abasic PNA probe.

### Spin-Tube: SMART-C-Biotin dynamic incorporation reaction on membranes and colorimetric readout

Reaction mixtures with a final volume of 300 µL were prepared by mixing: 45 µL of PCR products, 15 µL of SMART-C-Biotin (200 µM), 20 µL of sodium cyanoborohydride (NaBH_3_CN) - 15 mM in water, and 220 µL of SCD buffer. Protocol: All steps were performed using 300 µL and Spin-Tube devices were centrifuged to discard the solutions between each step. Membranes were incubated for 2 minutes at 45 °C with 300 µL of SCD buffer. The reaction mixture was added and incubated at 45 °C for 60 minutes. Upon completion of the incubation, three post-reaction washing steps were carried out using reagent I (pre-heated at 45 °C). This was followed by a blocking step in which membranes were incubated with reagent II for 5 minutes at RT. The enzymatic reaction was carried out by incubating the membranes with reagent III at RT for 5 minutes. Four post-enzymatic reaction washing steps with reagent IV were carried out. The chromogen solution (reagent V) was added to the membranes and left at 41 °C for 5 minutes. Finally, three post-chromogen washing steps with reagent VI were carried out. Membranes were able to be analyzed.

### Sensitivity and specificity of the assay

To assess the specificity of the assay, four tests were carried out: (1) one in which the PCR contained water instead of gDNAs and (2) another one in which 20 ng of human gDNA was used as template; and two more tests in which 20 ng of human gDNA were mixed with other 20 ng of each parasite (Fig. 5). On the other hand, for the sensitivity study, a range of PCR amplification reactions were performed using decreasing amounts of starting *T*. *cruzi* gDNA. Six different concentration points (10-fold dilutions) plus a negative control (water) were used in triplicate. All PCR amplification products were analyzed by capillary electrophoresis to determine the amount, if any, of amplicon had been generated (Fig. 5 for specificity and Fig. 6 and Table S1 for sensitivity). Expected size of amplicons were detected when the PCR was performed using the four highest amount of parasitic gDNAs while no bands were detected neither when using just human gDNA nor when using 0.1 and 0.01 pg/µL (Fig. 6B). Negative control (water) has not shown any signal, being truly a negative and so being effectively called as parasite free.

### *L*. *major* gDNA spike-in experiments on blood

DNA was extracted from 200 µL of human whole blood samples using QIAamp DNA Blood mini kit (Qiagen, Germany) following manufacturer’s guidelines. After proteinase K treatment, two samples were spiked with 10 and 0.1 ng of *L*. *major* gDNA. Then, the next steps of the DNA extraction protocol were followed as recommended. Samples were eluted in 30 µL of elution buffer AE. DNA was quantified with NanoDrop ND-1000 spectrophotometer version 3.3.1. (Thermo Fisher Scientific). PCR reactions were performed with a fix amount of DNA, 450 ng. After that, PCR were analyzed by capillary electrophoresis (using the Agilent 2100 Bioanalyzer and DNA 1000 Kit) and by the Spin-Tube.

## Conclusions

Our previous SNF sequence analysis by mass-based assay (MALDI-ToF) for *trypanosomiasis* identification has been further developed onto a new ultra-low-cost, easy and fast to use (~3 hours/test) Spin-Tube device. This novel device was designed to combine the dynamic chemical approach for nucleic acid reading (ChemNAT technology) with a colorimetric method in a plastic column and nylon membrane device (Spin-Tube). The Spin-Tube accurately distinguish and identify *Chagas disease* vs. *Leishmaniasis*. The test consists of a singleplex PCR to amplify a highly conserved sequence of DNA, that encodes the RNA component of the large ribosome subunit containing SNFs from the two different parasite species under interrogation. Amplicon identification to single base resolution was achieved. Dynamic chemistry enables preformans in a simple Spin-Tube. The assay allows for a naked-eye read-out of the unique colorimetric patterns coming for sample analysis. Clinical treatment decisions can be made without any ancillary equipment. The proposed Spin-Tube assay not only allows the detection of the presence of *Trypanosomatid* pathogens, but also differential diagnosis of *Leishmaniasis* vs. *Chagas disease*. Multiplexing is achieved by coupling various target-specific abasic probes onto nylon membranes using different array layouts and patterns. Incorporation of SMART-Biotin only into target sequences ensure high specificity. Clinically relevant sensitivity was obtained using our amplification and detection platform, down to a level of detection of 0.001 ng/µL (8.7 copies per µL) of gDNA from pathogen. Moreover, 0.1 ng of *L*. *major* gDNA was successfully detected when spiked-in with human blood samples. The assay has demonstrated a clinically relevant sensitivity and specificity^44^. A remarkable achievement was for the first time to succeed in the direct detection of ribosomal RNA. It opens up the possibility for direct detection of *Trypanosomatids* from biological fluids without any pre-amplification or pre-labelling of target nucleic acids. This breakthrough provides a prototype assay for an innovative PCR free product with many inherent benefits such as lower cross-contamination risk, simplification of protocol, reduction of time-to-results, significantly lower cost, to insure far lower risk of assay result errors. Concluding, we believe that the Spin-Tube developed by our group provides an accurate tool conbianing high sensitivity and specificity, permitting rapid identification and differential diagnosis of *Chagas disease* and *Leishmaniasis.* Its clinical value will be an improved patient monitoring and therapeutic decision making. The Spin-Tube opens-up the promise of repertoire of assays for other infectious diseases, such as malaria and tuberculosis.

## Supplementary information

Supplementary information

## Data Availability

All data and information are available upon request.
