# Supplementary material for: A colorimetric strategy based on dynamic chemistry for direct detection of Trypanosomatid species
Source: Sci Rep. 2019 Mar 6;9:3696. doi: 10.1038/s41598-019-39946-0 (PMC6403333; doi:10.1038/s41598-019-39946-0)
Supplement: Supplementary file 1 — Supplementary information [file 41598_2019_39946_MOESM1_ESM.docx]

**A colorimetric strategy based on dynamic chemistry for direct detection of Trypanosomatid species**

Mavys Tabraue-Chávez^a,^ ^†^, María Angélica Luque-González^b, c, †^, Antonio Marín-Romero^a, b, c^, Rosario María Sánchez-Martín^b, c^, Pablo Escobedo-Araque^d^, Salvatore Pernagallo^a, e, *^, Juan José Díaz-Mochón^a, b, c *^.

a. DestiNA Genomica S.L. Parque Tecnológico Ciencias de la Salud (PTS), Avenida de la Innovación 1, Edificio BIC, 18016 Armilla – Granada, Spain.

b. GENYO. Centre for Genomics and Oncological Research: Pfizer / University of Granada / Andalusian Regional Government. PTS Granada **-** Avenida de la Ilustración, 114 - 18016 Granada, Spain.

c. Dep. Medicinal and Organic Chemistry, School of Pharmacy, University of Granada, Campus Cartuja s/n, 18071 Granada, Spain.

d. ECsens, CITIC-UGR, Department of Electronics and Computer Technology, University of Granada, Campus Aynadamar, 18071 Granada, Spain.

e. DestiNA Genomics Ltd. 7-11 Melville St, Edinburgh EH3 7PE, United Kingdom. \

† These authors contributed equally to this work.

* Authors to whom correspondence should be addressed: [salvatore@destinagenomics.com](mailto:salvatore@destinagenomics.com) and [juanjose.diaz@genyo.es](mailto:juanjose.diaz@genyo.es).

**Nylon-membranes and Spin-Tube prototypes**

**
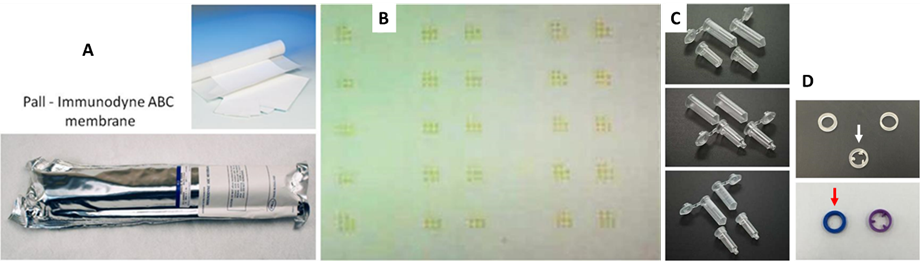
**

**Fig. S1.** A) Nylon membrane: Immunodyne^®^ ABC Membrane in roll format; B) Spotting probes on nylon membrane using PersonalArrayerTM 16 (CapitalBio Corporation, China); C) Different models of possible centrifuge collection tube and internal column for the Spin-Tube; D) Different models of a pressure plastic-rings either in color or transparent and with different shapes (the arrows indicate the plastic-rings used for this study).

**Abasic PNA probes sequences and monomers structures**

| **Probe code** | **DGL probe sequence (N-C)** |
| --- | --- |
| Abasic PNA_1_ | ## C C A **T*** A C T **T*** C ***GL*** C **T*** C A **C*** G A T |
| Abasic PNA_2_ | ## C **C*** A G A A A **C*** ***GL*** A G A G G **C*** A **C*** G |

**Fig. S2.** Sequences of abasic PNA probes. They have a free amino pegylated N-terminal (##). In black: neutral PNA monomers (X); in green: chiral and negatively charged monomers which contain propanoic acid residues at the γ-position of the PNA monomers (X*). In orange: chiral and negatively charged abasic PNA monomer (*GL*). NB: nitrogenous base.

**SMART-C-Biotin characterization**

**Fig. S3.** Structure of the biotin labelled aldehyde Cytosine (SMART-C-Biotin) for detection using colorimetric reporter system (in Fig. S4 and S5 respectively HPLC and MALDI-TOF MS characterization).


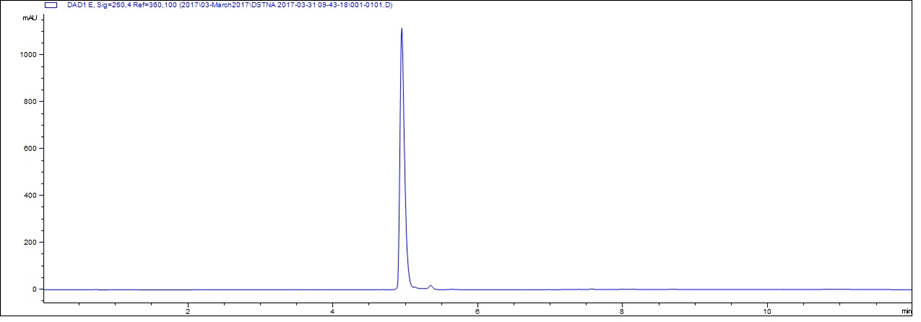


**Figure S4.** HPLC characterization. The purity of SMART-C-Biotin was greater than 98%.

**
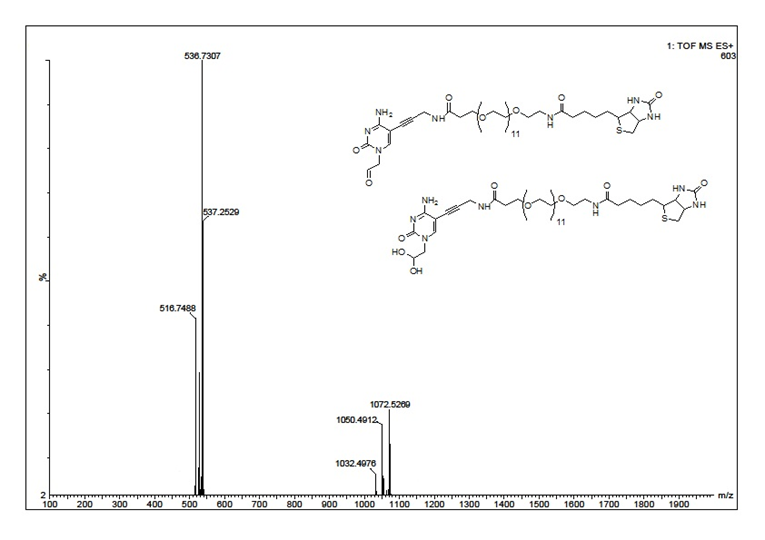
**

**Figure S5**. Chemical structure of SMART-C-Biotin, which bears a dodecylethylenglycol (PEG_12_) spacer. ES+-TOF MS of SMART-C-PEG6-Biotin, m/z 516.7 ([M+2H]^2+^), 536.7 ([M+H_2_O+H+Na]^2+^), 1032.4 ([M+H]^+^), 1050.4 ([M+H_2_O+H]^+^), 1072.4 ([M+H_2_O+Na]^+^). HRMS calcd. for C_46_H_78_N_7_O_17_S ([M+H]^+^) 1032.5175, found 1032.5137.

**Study of sensitivity**

| **ng of gDNA** | **ng/µL of gDNA** | **Copies/µL of gDNA** | **ng/µL of amplicon** |
| --- | --- | --- | --- |
| 50 | 1 | 8707 | 3.72 |
| 5 | 0.1 | 870.7 | 2.33 |
| 0.5 | 0.01 | 87.07 | 1.73 |
| 0.05 | 0.001 | 8.707 | 0.57 |
| 0.005 | 0.0001 | 0.87 | 0.10 |
| 0.0005 | 0.00001 | 0.087 | --- |
| --- | water | water | --- |

**Table S1**: First column shows the amount of gDNA (ng) used for each PCR reaction. This amount is expressed as concentration (ng/μL) in the second column and as number of copies per μL in the third column (assuming a gDNA length of 106.4 Mb^1^). Fourth column shows the concentration (ng/μL) of PCR product measured by Agilent 2100 Bioanalyzer.

**Relative signal intensity determination**

Although the Spin-Tube is essentially a qualitative test, relative signal intensities of the four positive membranes (from 50 to 0.05ng of gDNA) were calculated. Membranes pictures were analyzed with Image J software. A fixed circle template was used to measure the signal intensity for membrane background, biotin markers, PNA_1_ and PNA_2_. Five measurements were taken for membrane background signal, three for biotin markers and three for each abasic PNA probe. A mean value for each set of measurements was calculated by subtracting the background signal. Finally, considering the biotin markers signal as 100% of intensity for that specific membrane, the relative signal intensity for each probe PNA_1_ and PNA_2_ was calculated (Table S2). Because of the inter-membranes signal variability, these data have to be calculated individually; as a result, expressing signal relative intensities might be more accurate than an exact number. These relative intensities has been summarized in a graphic (Figure S6) in which the relative signal intensity of each set of spots has been represented *versus* the amount of templating gDNA used in the PCR reactions. It can be observed that PNA_1_ has a lower efficiency providing lower signal intensities. Moreover, whereas relative signal intensity seems to decrease as the amount of templating gDNA used in the PCR decrease, the signal obtained for PNA_2_ seems to maintain more or less stable regardless the amount of starting templating material.

| **Amount of gDNA (pg) used in PCR** | **PNA_1_ Relative Intensity (%)** | **PNA_2_ Relative Intensity (%)** |
| --- | --- | --- |
| 50000 | 33.92 | 32.01 |
| 5000 | 26.43 | 34.37 |
| 500 | 19.26 | 24.82 |
| 50 | 14.42 | 23.80 |

**Table S2:** Amount of gDNA used as template for the PCR amplification reactions and relative signal intensities of each abasic PNA probe after the incorporation of SMART-C-Biotin and the color development assay.

**Figure S6:** Signal relative intensity (%) *vs*. the amount of gDNA (pg) used as template for the PCR. As the amount of template decreases, the signal intensity decreases.

**Capillary electrophoresis of total and fragmented RNA**


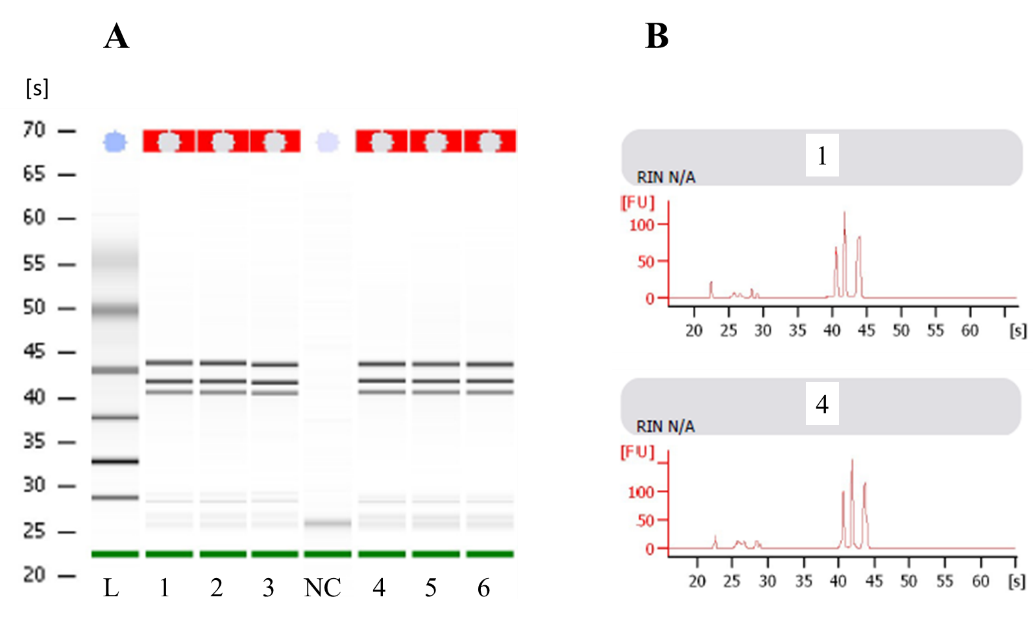


**Figure S7.** A) Agilent Bioanalyzer 2100 Gel-like images extrapolated from the capillary electrophoresis of total RNA samples. L: ladder; 1-3: Total RNA extracted from *L. major*. NC: Control (water); 4-6: Total RNA extracted from *T. cruzi*. Three bands are observed which correspond to the ribosomal subunits confirming thus the good quality of the extracted RNA. B) The fluorescence plots of total RNA extracted respectively from *L. major* (1) and *T. cruzi* (4). Both plots show three peaks corresponding with the expected trypanosomatids total RNA peak pattern[^2^](#_ENREF_2).


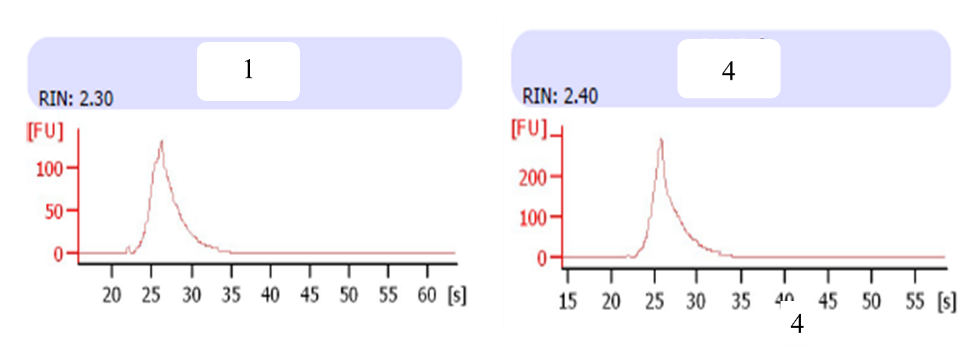


**Figure S8**. Agilent 2100 bioanalyzer and RNA 6000 Pico Kit analysis of total RNA fragmentation. Fluorescence plots of: 1) *L. major* and (4) *T. cruzi*. In both samples there was a decreasing ratio of ribosomal bands with the three peaks of the total RNA disappearing and confirming that total RNA was succesfully fragmented.

**References**

1 El-Sayed, N. M. *et al.* The genome sequence of Trypanosoma cruzi, etiologic agent of Chagas disease. *Science* **309**, 409-415, doi:10.1126/science.1112631 (2005).

2 Lambertz, U. *et al.* Small RNAs derived from tRNAs and rRNAs are highly enriched in exosomes from both old and new world Leishmania providing evidence for conserved exosomal RNA Packaging. *BMC genomics* **16**, 151, doi:10.1186/s12864-015-1260-7 (2015).
